# My burning issues in the neoadjuvant treatment for breast cancer

**DOI:** 10.1007/s12254-017-0378-5

**Published:** 2017-12-22

**Authors:** Elisabeth S. Bergen, Rupert Bartsch

**Affiliations:** 1 Comprehensive Cancer Center, Vienna, Austria; 2 Department of Medicine 1, Clinical Division of Oncology, Medical University of Vienna, Waehringer Guertel 18–20, 1090 Vienna, Austria; 3 German Breast Group, Neu-Isenburg, Germany

**Keywords:** Neoadjuvant therapy, Breast cancer

## Abstract

A combination of anthracyclines and taxanes remains the standard of care for neoadjuvant chemotherapy (NACT) resulting in increased breast conservation rate (BCR) and decreased recurrence rate [1]. Whether pathological complete response (pCR) could be an appropriate surrogate parameter for long-term survival is still a matter of debate. In patients with triple-negative breast cancer (TNBC) and HER2-positive breast cancer (BC), a six to nine times higher risk for relapse has been reported if no pCR was achieved [2, 3]. Within these aggressive subtypes the strongest association between pCR and long-term outcome could be observed [4]. However, a pooled analysis of recently conducted trials could only identify pCR as a surrogate endpoint for improved event-free survival (EFS) and overall survival (OS) on an individual patient level as opposed to the trial level [5]. Even in TNBC, demonstrating that an increased pCR converts into a significant survival benefit would require a study population markedly larger than calculated for previously conducted trials [6, 7].

## Triple negative subtype

About one third of patients with triple-negative breast cancer (TNBC) treated with standard NACT achieve pCR. Preclinical trials stated TNBC to be more sensitive to interstrand crosslinking agents such as platinum salts due to deficiencies in the BRCA-associated DNA repair mechanism [8]. Especially in *BRCA1*-mutated patients treated with carboplatin as part of NACT, pCR rates of up to 75% could be reached [9]. So far, five randomized phase II and one phase III trial addressed the use of carboplatin as part of NACT for patients with TNBC (Table 1):

**Table 1:** Phase II and III trials investigating the addition of carboplatin to neoadjuvant chemotherapy regimens for triple-negative patients; in studies including other subtypes only the triple-negative population is quoted

Study	Phase	Reference	Chemotherapy backbone	Experimental therapy	pCR
*GEICAM/2006-03*	II	Alba E et al. [10]	EC × 4 followed by D × 4	arm 1: D arm 2: D + Cb AUC 6	*p = 0.61*
*GeparSixto*	II	von Minckwitz et al. [11]	wPac + NPLD + Bev for 18 weeks	arm 1: + Cb AUC 1.5 arm 2: − Cb	*p = 0.015*
*CALGB 40603*	II	Sikov WM et al. [12]	wPac × 12 followed by ddAC × 4	arm 1: wPac arm 2: wPac + Bev arm 3: wPac + Cb AUC 6 × 4 arm 4: wPac + Bev + Cp	*p = 0.0029*
*WSG: ADAPT*	II	Gluz O et al. [13]	nab-Pac × 12	arm 1: nab-Pac + Cb arm 2: nab-Pac + Gem	*p < 0.001*
*i-SPY 2*	Generic trial platform	Rugo H et al. [14]	wPac × 12 followed by ddAC × 4	arm 1: wPac arm 2: wPac + pla A arm 3: wPac + pla B arm 4: wPac + Cp AUC 6 + Veli	*88% predicted probability*
*BRIGHTNESS*	III	Geyer CE et al. [15]	wPac × 12 followed by ddAC × 4	arm 1: wPac + Cb AUC 6 + Veli arm 2: wPac + Cb + pla arm 3: wPac + pla + pla	*arm 1 vs arm 3: p < 0.001* *arm 1 vs arm 2: p = 0.36*

*EC* epirubicin/cyclophosphamide; *D* docetaxel; *Cb* carboplatin; *wPac* weekly paclitaxel; *NPLD* non-pegylated liposomal doxorubicin; *Bev* bevacizumab; *AUC* area under the curve; *ddAC* doxorubicin plus cyclophosphamide once every 2 weeks; *nab-Pac* nab-paclitaxel; *Gem* gemcitabine; *pla* placebo (A and B); *Veli* veliparib

The *GEICAM* trial represents the only negative trial so far; here, a nonsignificant drop of pCR with the addition of carboplatin to docetaxel after an anthracycline-based regimen was reported (*p =* 0.61) [10]. In the triple-negative population of *GeparSixto*, the addition of weekly carboplatin to weekly paclitaxel, non-pegylated liposomal doxorubicin and three-weekly bevacizumab increased pCR rates in breast/axilla from 43 to 57% (*p =* 0.015) [11]. Of note, the control arm did not include cyclophosphamide. In the *CALGB 40603* trial, 54% compared to 41% of patients had pCR with the addition of carboplatin when added to a standard chemotherapy backbone of paclitaxel followed by doxorubicin plus cyclophosphamide once every two weeks (*p =* 0.0029) [12]. The *ADAPT* triple-negative trial revealed a statistically significant benefit of carboplatin compared to gemcitabine when added to nab-paclitaxel (*p <* 0.001) [13]. Finally, the generic trial platform *i-SPY 2* compared paclitaxel with or without an experimental combination of carboplatin and the PARP-inhibitor veliparib followed by doxorubicin and cyclophosphamide in 116 patients. Carboplatin and veliparib graduated with 88% predicted probability of success in a phase 3 study [14].

*BRIGHTNESS* was the first randomized, placebo-controlled phase III trial investigating carboplatin for NACT; results were presented at the 2017 ASCO annual meeting. Triple-negative patients were randomized 1:1:2 to paclitaxel either alone or with carboplatin or with the combination of carboplatin and veliparib followed by doxorubicin plus cyclophosphamide. The addition of carboplatin and veliparib significantly increased pCR rates (53.2% vs 31.0%, *p <* 0.001), while addition of veliparib to carboplatin and paclitaxel did not demonstrate further improvement (53.2% vs 57.5%, *p =* 0.36) [15].

In all of the trials, carboplatin was associated with significantly increased grade 3/4 side effects with consecutive treatment discontinuations which clearly has to be weighed against improved activity. Summarizing current data, the benefit of carboplatin in the neoadjuvant setting could be clearly shown. Large prospective studies are needed to compare doses and schedules of carboplatin (AUC 1.5 or 2 weekly vs AUC 5 or 6 every 3 weeks) as well as chemotherapy backbones to reduce side effects and improve tolerability.

## HER2-positive subtype

Achieving a pCR in HER2-positive patients results in a significantly better event-free and overall survival as shown in the Collaborative Trials in Neoadjuvant Breast Cancer (*CTNeoBC*) pooled analysis [5]. Dual targeting the HER2 receptor with trastuzumab and pertuzumab in combination with docetaxel results in a significantly higher proportion of patients achieving pCR compared to trastuzumab plus docetaxel alone *(p =* 0.014) [16]. Importantly, dual blockade does not increase the rate of symptomatic left ventricular systolic dysfunction as shown in the *TRYPHAENA* study [17]. Several trials also reported increased pCR rates with the vertical blockade of trastuzumab and lapatinib compared to trastuzumab in combination with paclitaxel. A limiting factor of all neoadjuvant trials investigating this combination clearly was the increase in toxicity and treatment discontinuations [18–20]. Within all HER2-positive trials, a lower pCR rate was obtained in estrogen receptor(ER)-positive patients highlighting the distinct tumor biology of this subtype and the need for predictive biomarkers.

Of note, in the *NOAH* study, the pCR increase from 19 to 38% by adding trastuzumab to NACT was reflected in improved event-free survival (EFS) since it was the only neoadjuvant trial powered for this endpoint [21].

## Luminal subtype

Currently, NACT in luminal patients remains on option preferentially for large and/or highly proliferative tumors as it may be less effective in estrogen receptor (ER)-positive compared to ER-negative tumors [22].

So far, two randomized phase II trials compared the efficacy and tolerability of preoperative endocrine therapy (ET) and chemotherapy in luminal populations. Semiglazov et al. randomized 239 patients with IIa to IIIb luminal tumors to four cycles of anthracyclines and taxanes for 12 weeks or ET with anastrozole or exemestane for 3 months. Beside the limitation of missing HER2 status and the suboptimal chemotherapy backbone, no differences in clinical or pathological response could be determined between treatment groups [23]. In another randomized phase II trial including premenopausal patients, higher clinical response rates could be observed with an anthracycline–cyclophosphamide combination followed by taxanes compared to exemestane (in combination with goserelin if patients were premenopausal) (66% vs 48%; *p =* 0.075). In contrast, in the subpopulation of patients with a low proliferation rate, response rates had been similar in both arms (63% vs 58%; *p =* 0.74) [24].

Therefore, neoadjuvant ET may be a feasible and well-tolerated option for postmenopausal women with either low proliferating tumors, comorbid conditions and/or advanced age.

## Conclusions

Neoadjuvant instead of adjuvant therapy is the preferred treatment approach for clinical stage II and III TNBC and HER2-positive BC as stated by the St. Gallen Consensus Conference [25]. pCR as primary endpoint allows objective evaluation of treatment sensitivity. On an individual patient level, pCR correlates with improved long-term outcome.
